# Supplementary material for: Neutralizing antibodies reveal cryptic vulnerabilities and interdomain crosstalk in the porcine deltacoronavirus spike protein
Source: Nat Commun. 2024 Jun 22;15:5330. doi: 10.1038/s41467-024-49693-0 (PMC11193727; doi:10.1038/s41467-024-49693-0)
Supplement: Supplementary file 1 — Supplementary Information [file 41467_2024_49693_MOESM1_ESM.pdf]

Neutralizing antibodies reveal cryptic vulnerabilities and interdomain crosstalk in the porcine deltacoronavirus spike protein

Wenjuan Du[1#], Oliver Debski-Antoniak[1#], Dubravka Drabek[2,3], Rien van Haperen[2,3], Melissa van Dortmondt[1], Joline van der Lee[1], Ieva Drulyte[4], Frank J.M. van Kuppeveld[1], Frank Grosveld[2,3], Daniel L. Hurdiss[1]* and Berend-Jan Bosch[1]*

[1]Virology Section, Infectious Diseases and Immunology Division, Department of Biomolecular Health Sciences, Faculty of Veterinary Medicine, Utrecht University, Utrecht, the Netherlands

[2]Department of Cell Biology, Erasmus Medical Center, Rotterdam, the Netherlands

[3]Harbour BioMed, Rotterdam, the Netherlands

[4]Thermo Fisher Scientific, Materials and Structural Analysis, Eindhoven, the Netherlands

Contributions: # These authors contributed equally.

*Corresponding authors: Daniel L. Hurdiss; d.l.hurdiss@uu.nl and Berend-Jan Bosch; b.j.bosch@uu.nl

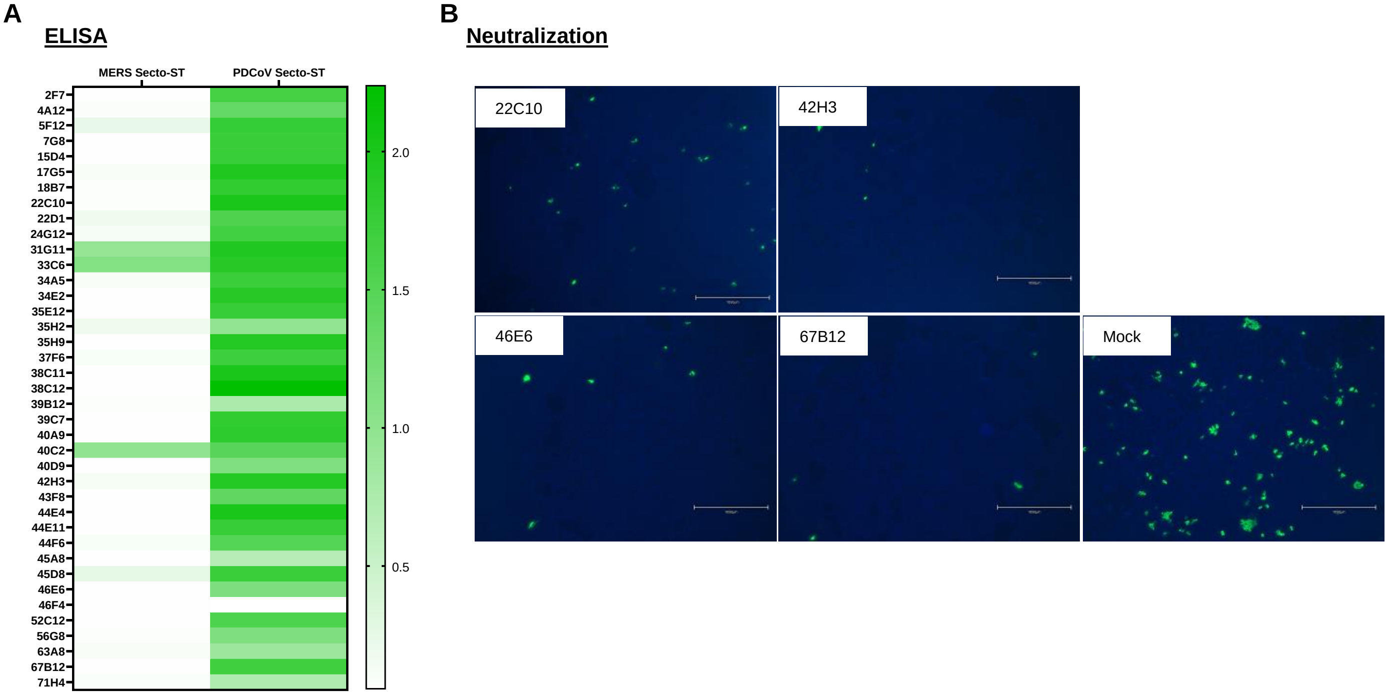

Fig.S1 Screening of H2L2 antibodies for binding and neutralization activities. (**A**) ELISA binding signal of H2L2 hybridoma culture supernatants (diluted 10-fold) to PDCoV S ectodomain (Secto). MERS-CoV Secto was included to exclude antibodies binding to the GCN4 trimerization tag and affinity purification Strep-tag (ST) fused to the C-terminus of both PDCoV and MERS-CoV Secto. Mean values from two independent experiments are presented. (**B**) Neutralization of PDCoV by H2L2 mAbs. PDCoV (600 TCID50) was preincubated with purified H2L2 mAbs (10 µg/ml) at room temperature for 1 hour before overlaying onto confluent monolayers of Huh7 cells. PDCoV-infected cells were stained by using anti-nucleocapsid protein (NP) mAb 15 hpi. Experiments were performed two times, one representative experiment is shown. Scale bar represents 1050 µm.

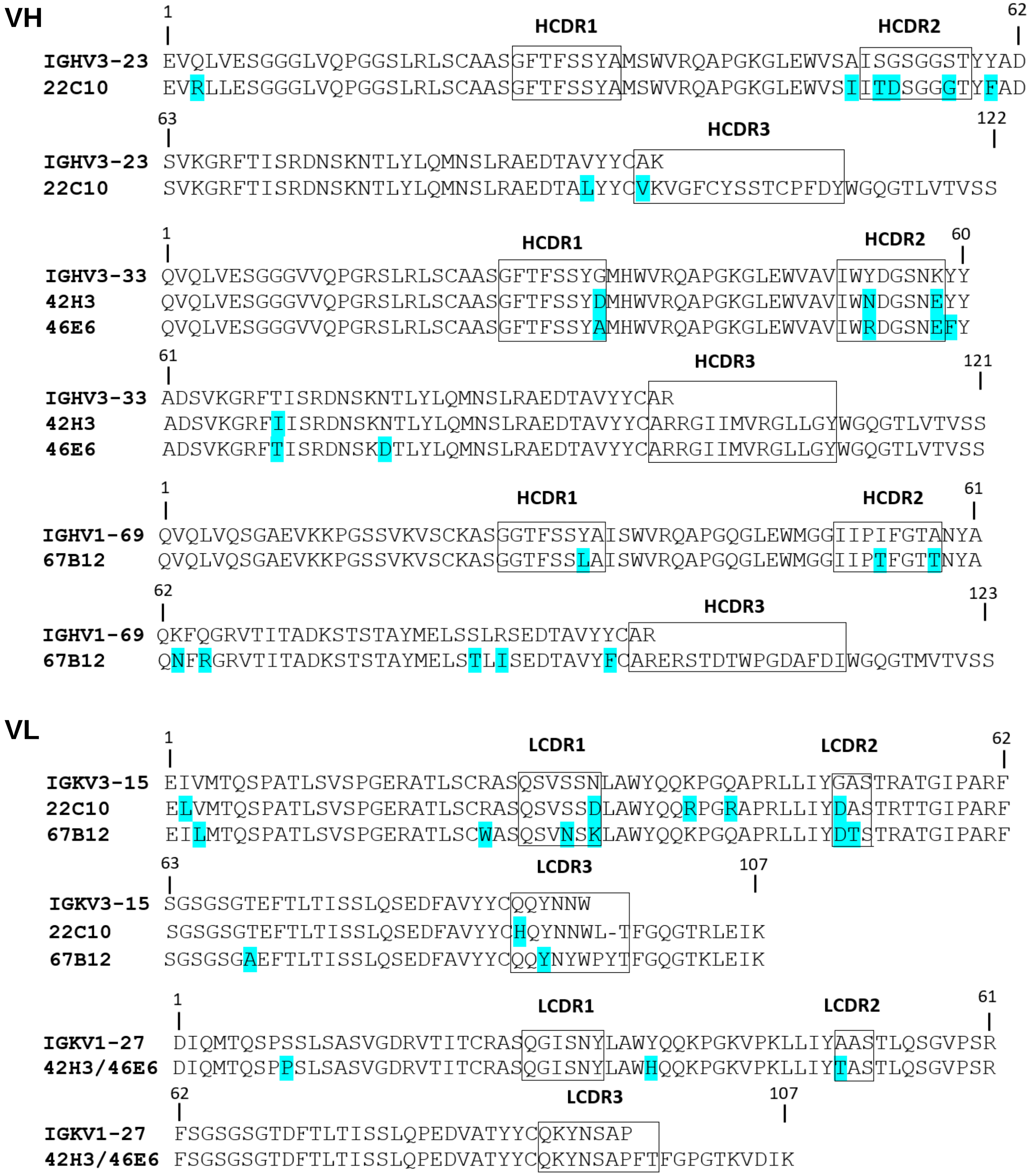

Fig.S2 Sequence alignment of VH (upper panel) and VL (lower panel) domains of four neutralizing PDCoV S mAbs and the human germline sequence. Somatic hypermutations in each antibody sequence are highlighted in turquoise, and the complementary determining regions (CDRs) are boxed.

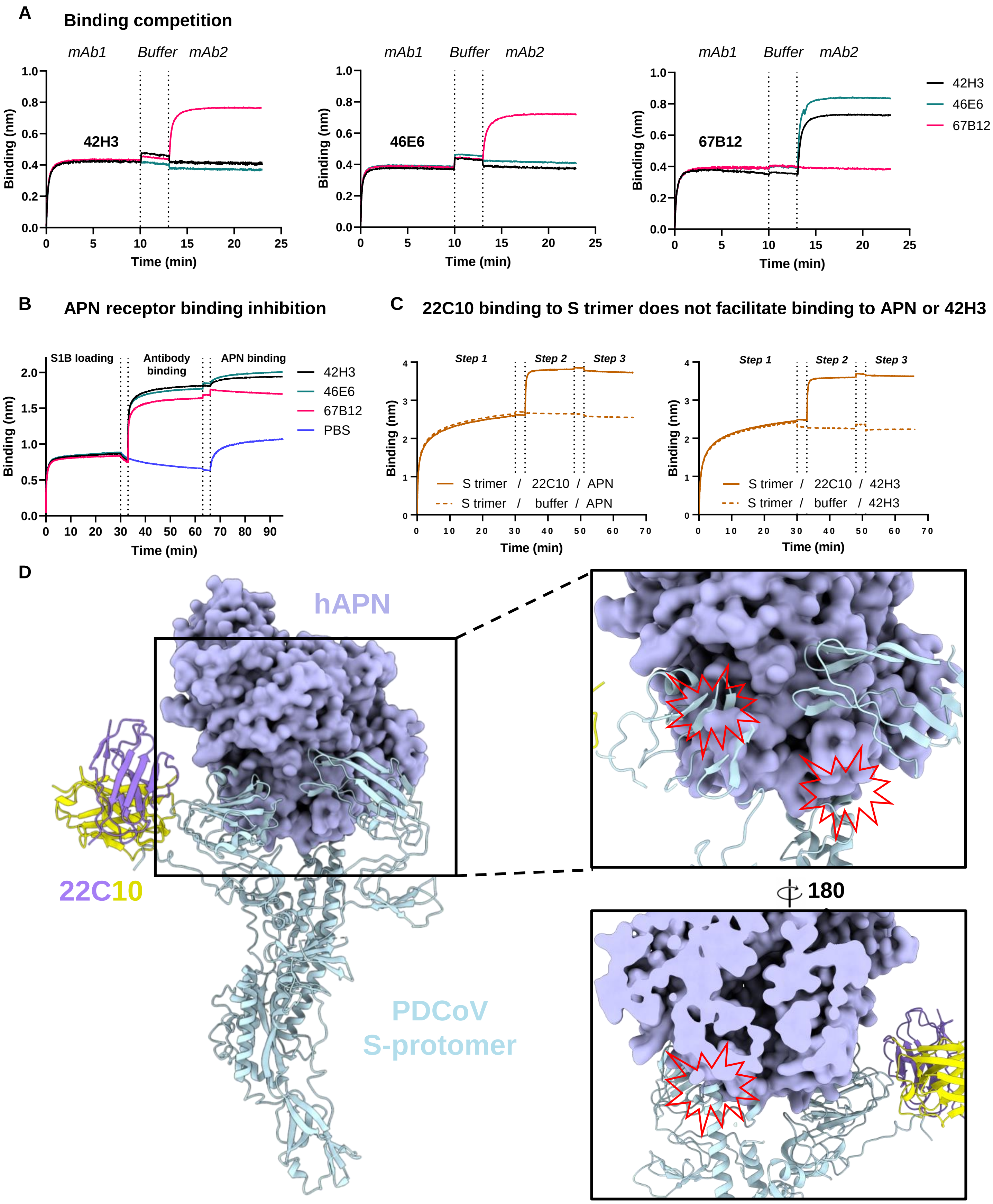
A
Binding competition
mAb1
Buffer
mAb2
42H3
46E6
67B12
Binding (nm)
Time (min)
B
APN receptor binding inhibition
S1B loading
Antibody binding
APN binding
42H3
46E6
67B12
PBS
C
22C10 binding to S trimer does not facilitate binding to APN or 42H3
Step 1
Step 2
Step 3
S trimer / 22C10 / APN
S trimer / buffer / APN
S trimer / 22C10 / 42H3
S trimer / buffer / 42H3
D
hAPN
22C10
PDCoV S-protomer
180

Fig.S3 (**A**) Binding competition between three S1B-reactive mAbs using Bio-Layer Interferometry (BLI). Biosensor-immobilized PDCoV-S1 antigen was allowed to bind with a specific human PDCoV S mAb (referred to as mAb 1) and then exposed to a second mAb (mAb 2) after a 3 min wash step using PBS. (**B**) BLI-based assessment of APN receptor binding inhibition. Strep-tagged PDCoV S1B was immobilized onto a Protein A biosensor previously bound with anti-Streptag mAb, followed by the binding of the indicated mAbs (50 µg/ml) for 30 min. To analyze whether the antibody bound S1B domain is still capable of interacting with the APN receptor, biosensors are immersed into wells containing chicken APN (150 µg/ml) for an additional 30 min. Experiments were carried out twice, with a representative experiment shown. (**C**) Binding of 22C10 to PDCoV spike trimer does not facilitate binding to APN or 43H3, as evaluated by BLI. Strep-tagged PDCoV S prefusion trimer was captured by anti-Streptag mAb bound on Protein A biosensor (Step 1). Subsequently biosensors were dipped into wells with either 22C10 present (50 µg/ml, solid line) or absent (dashed line) for 15min (Step 2), followed by incubation with chicken APN (150 µg/ml; Step 3; left panel) or 42H3 (50 µg/ml; Step 3; right panel) for an additional 30 min. Experiments were performed twice, with a representative experiment shown. (**D**) Superimposition of human APN/PDCoV S1B complex (PDB: 7VPQ) onto a protomer of the 22C10 bound PDCoV spike protein structure. Clashes between human APN and 22C10-bound PDCoV spike monomer are depicted with a red clash icon.

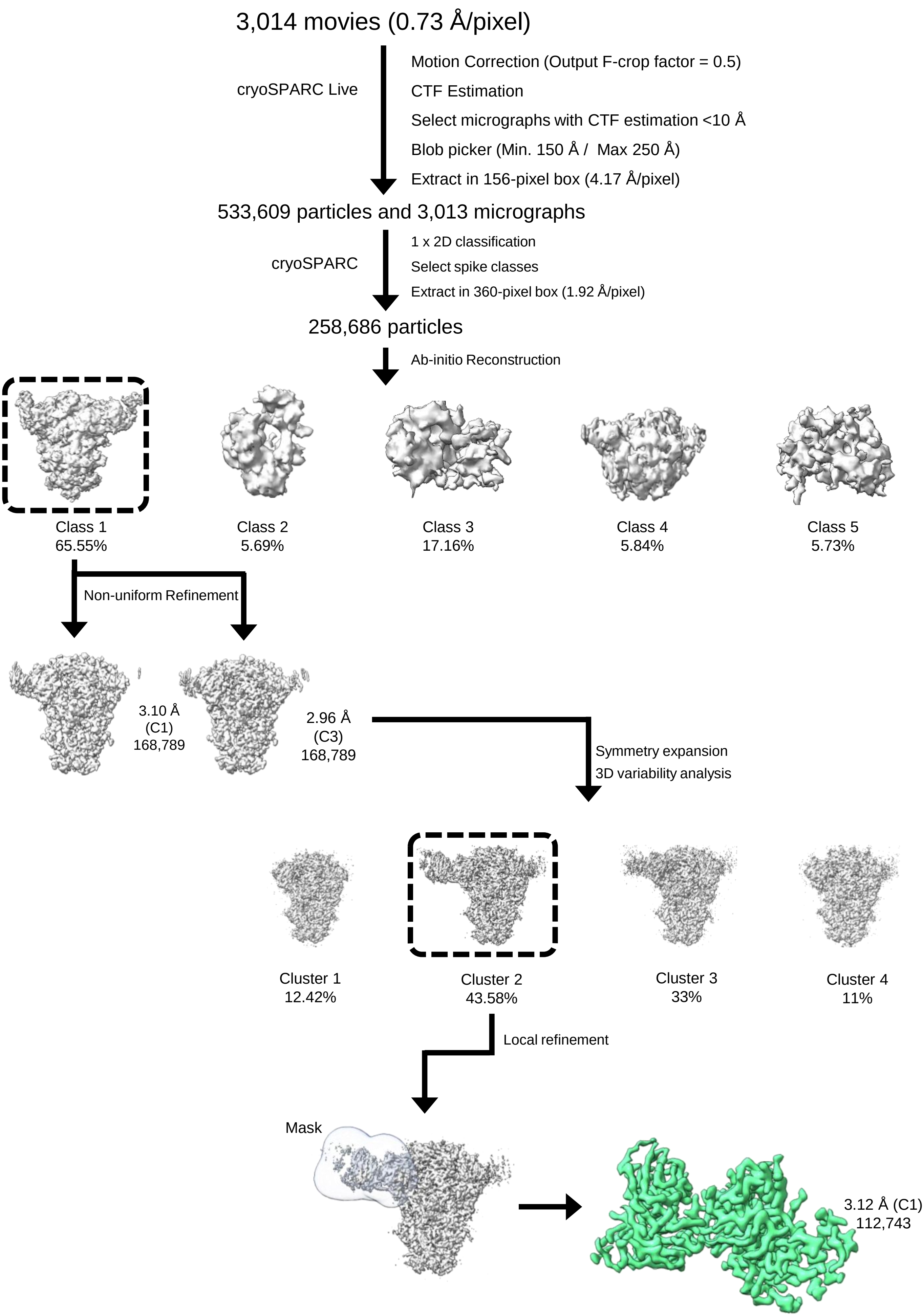

Fig.S4 Single-particle cryo-EM data processing pipeline for the PDCoV S-22C10 complex.

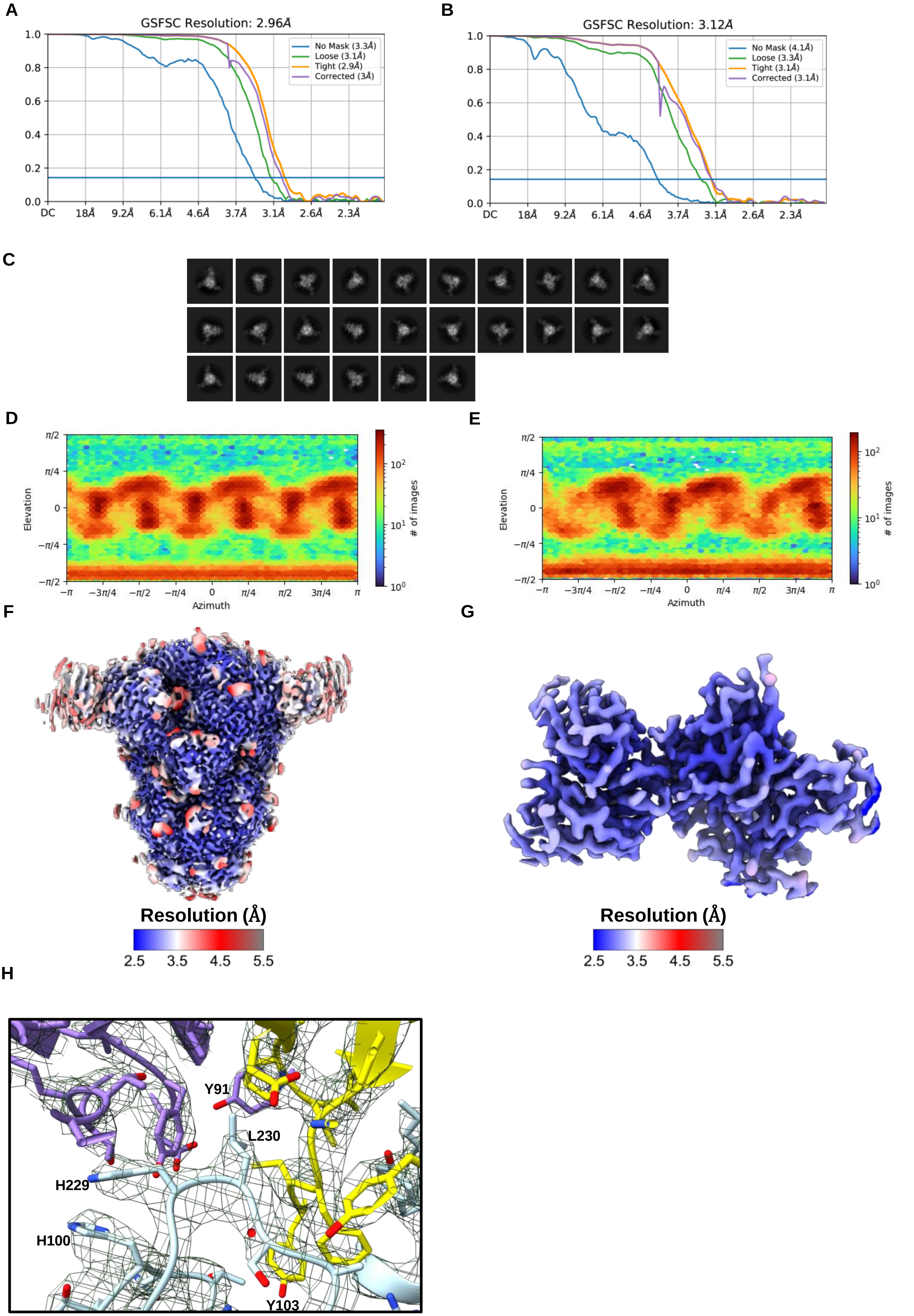
A
GSFSC Resolution: 2.96Å
No Mask (3.3Å)
Loose (3.1Å)
Tight (2.9Å)
Corrected (3Å)
B
GSFSC Resolution: 3.12Å
No Mask (4.1Å)
Loose (3.3Å)
Tight (3.1Å)
Corrected (3.1Å)
DC
18Å
9.2Å
6.1Å
4.6Å
3.7Å
3.1Å
2.6Å
2.3Å
C
D
E
Elevation
Azimuth
# of images
F
G
Resolution (Å)
2.5
3.5
4.5
5.5
H
Y91
L230
H229
H100
Y103

Fig.S5 Single-particle cryo-EM data processing for the PDCoV S-22C10 complex. (**A**) Gold-standard Fourier shell correlation (FSC) curve generated from the independent half maps contributing to the 2.9 Å global resolution density map of the PDCoV spike in complex with 22C10 Fab fragment. (**B**) Gold-standard Fourier shell correlation (FSC) curve generated from the independent half maps contributing to the 3.1 Å local refined density map of the PDCoV spike-22C10 complex paratope-epitope. (**C**) Representative 2D classes. (**D**) Angular distribution plot of the final global C3 refined EM density map. (**E**) Angular distribution plot of the local C1 refined EM density map. (**F**) Local resolution filtered EM density map for the C3 refined PDCoV spike-22C10 complex, colored according to local resolution which was calculated in CryoSPARC. (**G**) Local resolution filtered EM density map for the local refinement of the PDCoV spike-22C10 complex paratope-epitope, colored according to local resolution which was calculated in CryoSPARC. (**H**) Zoomed-in view of the interacting region of 22C10 and PDCoV S1A with the EM density of the local refinement shown as a black mesh.

```
                   1                                                   53
AHL45007.1         MQRALLIMTLLCLVRAKFADDLLDLLTFPGAHRFLHKPTRNSSSLYSRANNNF
AHN16220.1         MQRALLIMTLLCLVRAKFADDLLDLLTFPGAHRFLHKPTRNSSSLYSRANNNF
AML40790.1         MQRALLIMTLLCLVRAKFADDLLDLLTFPGAHRFLHKPTRNSSSLYSRANNNF
AML40604.1         MQRALLIMTLLCLVRAKFADDLLDLLTFPGAHRFLHKPTRNSSSLYSRANNNF
QZX45753.1         MQRALLIMTLLCLVRAKFADDLLDLLTFPGAHRFLHKPTSNSSSHYSRANN-F
AKC54442.1         MQRALLIMTLLCLVRAKFADDLLDLLTFPGAHRFLHKLTSNSSSLYSRANN-F
UVJ47552.1         MQRALLIMTLLCLVRAKFADDLLDLLTFPGAHRFLHKLTSNSSSLYSRANN-F
QWE80492.1         MQRALLIMTLLCLVRAKFADDLLDLLTFPGAHRFLHKLTSNSSSFYSRANN-F

                   54                                                 106
AHL45007.1         DVGVLPGYPTKNVNLFSPLTNSTLPINGLHRSYQPLMLNCLTKITNHTLSMYL
AHN16220.1         DVGVLPGYPTKNVNLFSPLTNSTLPINGLHRSYQPLMLNCLTKITNHTLSMYL
AML40790.1         DVGVLPGYPTKNVNLFSPLTNSTLPINGLHRSYQPLMLNCLTKITNHTLSMYL
AML40604.1         DVGVLPGYPTKNVNLFSPLTNSTLPINGLHRSYQPLMLNCLTKITNHTLSMYL
QZX45753.1         DVGVLPGYPTKNVNLFSPLTNSTLPINGLHRSYQPLMLNCLTKITNHTLSMYL
AKC54442.1         DVGVLPGYPTKNVNLFSPLTNSTLPINGLHRSYQPLMLNCLTKITNHTLSMYL
UVJ47552.1         DVGVLPGYPTKNVNLFSPLTNSTLPINGLHRSYQPLMLNCLTKITNHTLSMYL
QWE80492.1         DVGVLPGYPTENVNLFSPLTNSTLPINGLHRSYQPLMLNCLTKITNHTLSMYL

                   107                                                159
AHL45007.1         LPSEIQTYSCGGAMVKYQTHDAVRIILDLTATDHISVEVVGQHGENYVFVCSE
AHN16220.1         LPSEIQTYSCGGAMVKYQTHDAVRIILDLTATDHISVEVVGQHGENYVFVCSE
AML40790.1         LPSEIQTYSCGGAMVKYQTHDAVRIILDLTATDHISVEVVGQHGENYVFVCSE
AML40604.1         LPSEIQTYSCGGAMVKYQTHDAVRIILDLTTTDHISVEVVGQHGENYVFVCSE
QZX45753.1         LPSEIQTYSCGGAMVKHQTHDAVRIILDLTATDHISVEVVGQHGENYVFVCSE
AKC54442.1         LPSEIQTYSCGGAMVKYQTHDAVRIILDLTATDHISVEVVGQHGENYVFVCSE
UVJ47552.1         LPSEIQTYSCGGAMVKYQTHDAVRIILDLTVTHHISVEVVGQHGENYVFVCSE
QWE80492.1         LPSEIQTYSCGGAMVKYQTHDAVRIILDLTVTDHISVEVVGQRGENYVFVCSE

                   160                                                212
AHL45007.1         QFNYTTALHNSTFFSLNSELYCFTNNTYLGILPPDLTDFTVYRTGQFYANGYL
AHN16220.1         QFNYTTALHNSTFFSLNSELYCFTNNTYLGILPPDLTDFTVYRTGQFYANGYL
AML40790.1         QFNYTTALHNSTFFSLNSELYCFTNNTYLGILPPDLTDFTVYRTGQFYANGYL
AML40604.1         QFNYTTALHNSTFFSLNSELYCFTNNTYLGILPPDLTDFTVYRTGQFYANGYL
QZX45753.1         QFNYTTALHNSTFFSLNSELYCFTNNTYLGILPPDLTDFTVYRTGQFYANGYL
AKC54442.1         QFNYTTALHNSTFFSLNSELYCFTNNTYLGILPPDLTDFTVYRTGQFYANGYL
UVJ47552.1         QFNYTTALRKSTFFSLNSKLYCFTNNTYLGILPPDLTDFTVYRTGQFYANGYL
QWE80492.1         QFNYTTALHNSTFFSLNSELYCFTNNTYLGILPPDLTDFTVYRTGQFYANGYL

                   213                                                265
AHL45007.1         LGTLPITVNYVRLYRGHLSANSAHFALANLTDTLITLTNTTISQITYCDKSVV
AHN16220.1         LGTLPITVNYVRLYRGHLSANSAHFALANLTDTLITLTNTTISQITYCDKSVV
AML40790.1         LGTLPITVNYVRLYRGHLSANSAHFALANLTDTLITLTNTTISQITYCDKSVV
AML40604.1         LGTLPITVNYVRLYRGHLSANSAHFALANLTDTLITLTNTTISQITYCDKSVV
QZX45753.1         LGTLPITVNYVRLYRGHLSANSAHFALANLTDTLITLTNTTISQITYCDKSVV
AKC54442.1         LGTLPITVNYVRLYRGHLSANSAHFALANLTDTLITLTNTTISQITYCDKSVV
UVJ47552.1         LGTLPITVNYVRLYRGHLSANSAHFALANLTDTLITLTNTTISQITYCDKSVV
QWE80492.1         LGTLPITVNYVRLYRGHLSANSAHFALANLTDTLITLTNTTISQITYCDKSVV
```

Continued

```
                     266                                                  318
AHL45007.1           DSIACQRSSHEVEDGFYSDPKSAVRARQRTIVTLPKLPELEVVQLNISAHMDF
AHN16220.1           DSIACQRSSHEVEDGFYSDPKSAVRARQRTIVTLPKLPELEVVQLNISAHMDF
AML40790.1           DSIACQRSSHEVEDGFYSDPKSAVRARQRTIVTLPKLPELEVVQLNISAHMDF
AML40604.1           DSIACQRSSHEVEDGFYSDPKSAVRARQRTIVTLPKLPELEVVQLNISAHMDF
QZX45753.1           DSIACQRSSHEVEDGFYSDPKSAVRARQRTIVTLPKLPELEVVQLNISAHMDF
AKC54442.1           DSIACQRSSHEVEDGFYSDPKSAVRARQRTIVTLPKLPELEVVQLNISAHMDF
UVJ47552.1           DSIACQRSSHEVEDGFYSDPKSAVRARQRTIVTLPKLPELEVVQLNISAHMDF
QWE80492.1           DSIACQRSSHEVEDGFYSDPKSAVRARQRTIVTLPKLPELEVVQLNISAHMDF

                     319                                                  371
AHL45007.1           GEARLDSVTINGNTSYCVTKPYFRLETNFMCTGCTMNLRTDTCSFDLSAVNNG
AHN16220.1           GEARLDSVTINGNTSYCVTKPYFRLETNFMCTGCTMNLRTDTCSFDLSAVNNG
AML40790.1           GEARLDSVTINGNTSYCVTKPYFRLETNFMCTGCTMNLRTDTCSFDLSAVNNG
AML40604.1           GEARLDSVTINGNTSYCVTKPYFRLETNFMCTGCTMNLRTDTCSFDLSAVNNG
QZX45753.1           GEARLDSVTINGNTSYCVTKPYFRLETNFMCTGCTMNLRTDTCSFDLSAVNNG
AKC54442.1           GEARLDSVTINGNTSYCVTKPYFRLETNFMCTGCTMNLRTDTCSFDLSAVNNG
UVJ47552.1           GEARLDSVTINGNTSYCVTKPYFRLETNFMCTGCTMNLRTDTCSFDLSAVNNG
QWE80492.1           GEARLDSVTINGNTSYCATKPYFRLETNFMCTGCTMNLRTDTCSFDLSAVNNG

                     372                                                  424
AHL45007.1           MSFSQFCLSTESGACEMKIIVTYVWNYLLRQRLYVTAVEGQTHTGTTSVHATD
AHN16220.1           MSFSQFCLSTESGACEMKIIVTYVWNYLLRQRLYVTAVEGQTHTGTTSVHATD
AML40790.1           MSFSQFCLSTESGACEMKIIVTYVWNYLLRQRLYVTAVEGQTHTGTTSVHATD
AML40604.1           MSFSQFCLSTESGACEMKIIVTYVWNYLLRQRLYVTAVEGQTHTGTTSVHATD
QZX45753.1           MSFSQFCLSTESGACEMKIIVTYVWNYLLRQRLYVTAVEGQTHTGTTSVHATD
AKC54442.1           MSFSQFCLSTESGACEMKIIVTYVWNYLLRQRLYVTAVEGQTHTGTTSVHATD
UVJ47552.1           MSFSQFCLSTESGACEMKIIVTYVWKYLLRQRLYVTAVEGQTHTGTTSVHATD
QWE80492.1           MSFSQFCLSTESGACEMKIIVTYVWNYLLRQRLYVTAVEGQTHTGTTSVHATD

                     425                                                  477
AHL45007.1           TSSVITDVCTDYTIYGVSGTGIIKPSDLLLHNGIAFTSPTGELYAFKNITTGK
AHN16220.1           TSSVITDVCTDYTIYGVSGTGIIKPSDLLLHNGIAFTSPTGELYAFKNITTGK
AML40790.1           TSSVITDVCTDYTIYGVSGTGIIKPSDLLLHNGIAFTSPTGELYAFKNITTGK
AML40604.1           TSSVITDVCTDYTIYGVSGTGIIKPSDLLLHNGIAFTSPTGELYAFKNITTGK
QZX45753.1           TSSVITDVCTDYTIYGVSGTGIIKPSDLLLHNGIAFTSPTGELYAFKNITTGK
AKC54442.1           TSSVITDVCTDYTIYGVSGTGIIKPSDLLLHNGIAFTSPTGELYAFKNITTGK
UVJ47552.1           TSSVITDVCTDYTIYGVSGTGIIKPSDLLLHNGIAFTSPTGELYAFKNITTGK
QWE80492.1           TSSVITDVCTDYTIYGVSGTGIIKPSDLLLHNGIAFTSPTGELYAFKNITTGK

                     478                                             525
AHL45007.1           TLQVLPCETPSQLIVINNTVVGAITSSNSTENNRFTTTIVTPTFFYST
AHN16220.1           TLQVLPCETPSQLIVINNTVVGAITSSNSTENNRFTTTIVTPTFFYST
AML40790.1           TLQVLPCETPSQLIVINNTVVGAITSSNSTENNRFTTTIVTPTFFYST
AML40604.1           TLQVLPCETPSQLIVINNTVVGAITSSNSTENNRFTTTIVTPTFFYST
QZX45753.1           TLQVLPCETPSQLIVINNTVVGAITSSNSTENNRFTTTIVTPTFFYST
AKC54442.1           TLQVLPCETPSQLIVINNTVVGAITSSNSTENNRFTTTIVTPTFFYST
UVJ47552.1           TLQVLPCETPSQLIVINNTVVGAITSSNSTENNRFTTTIVTPTFFYST
QWE80492.1           TLQVLPCETPSQLIVINNTVVGAITSSNSTENNRFTTTIVTPTFFYST
```

Fig.S6 Alignment of PDCoV S1 protein sequences from viruses isolated across different countries. PDCoV S1 protein sequences were aligned from viruses obtained from the USA (GenBank accession codes: AHL45007.1 [used in this study], AHN16220.1, AML40790.1 and AML40604.1), China (QZX45753.1, AKC54442.1 and UVJ47552.1) or Haiti (QWE80492.1, human isolate). Amino acid variations from the strain used in this study are highlighted in turquoise.

A

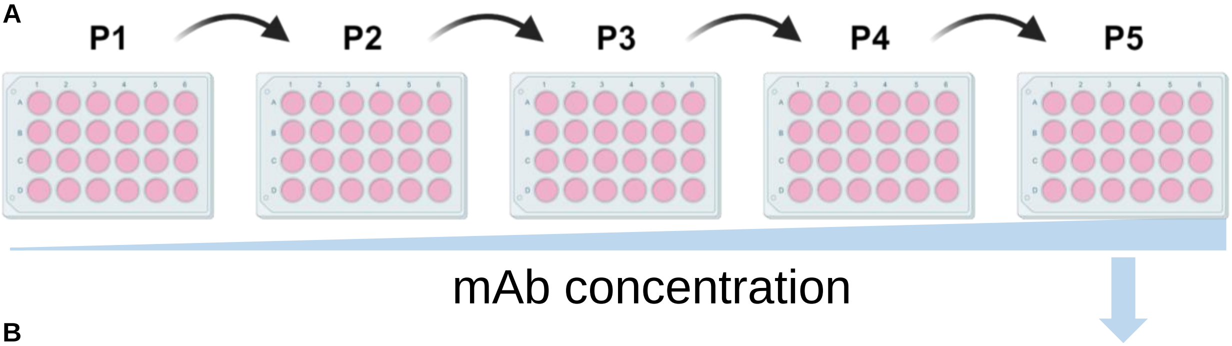

B

			S mutations identified in PDCoV following antibody selection				
	mAb	Clone	S1A	S1B		S2	
S1A binder	22C10	#1	S231F				
		#2	del232-234				
S1B binder	42H3	#1		S362R			
		#2			E410G		
	46E6	#1		D359A	E410Q		
		#2		S362R			
	67B12	#1				V709G	
		#2				I705S	
		#3				I705T	
		#4				I705T	Q1072K
		#5			N331T	I705T	Q1072K
	MOCK1						
	MOCK2						

Fig.S7 Spike mutations identified in PDCoV following antibody selection. (**A**) Antibody escape mutant viruses were generated by serial passaging of PDCoV on Huh7 cells in the presence of each of the four monoclonal antibodies 22C10, 67B12, 42H3 and 46E6. This figure was created with BioRender.com. (**B**) Spike genes of selected clonal viruses from limited dilution were sequenced at the indicated passages using viral RNA extracted from cell supernatants.

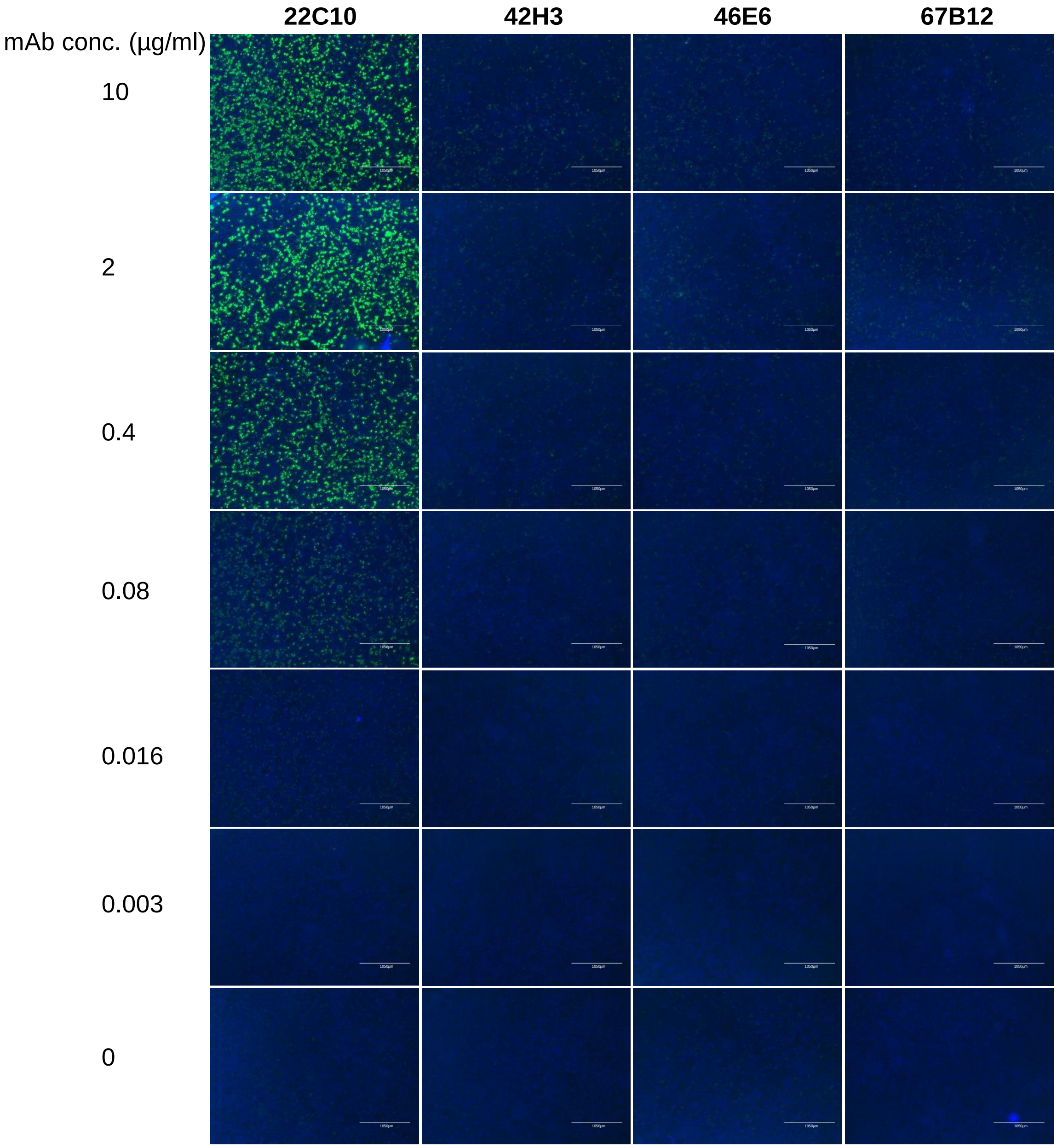

Fig.S8 Binding of antibodies to cell-surface expressed PDCoV S proteins. The full length PDCoV S protein was expressed in Huh7 cells by plasmid transfection. After fixation, cells were subjected to binding with serially diluted mAbs targeting epitopes that were found to be exposed (mAb 22C10) or occluded (mAbs 42H3, 46E6, 67B12) in the prefusion S trimer. Antibody binding was monitored by immunofluorescence assay. Cell nuclei in the overlay images were visualized by DAPI. Fluorescence images were recorded using EVOS M5000 Cell Imaging System (Thermo Fisher Scientific). Representative images are shown from n = 2 biological replicates. Scale bar represents 1050 µm.

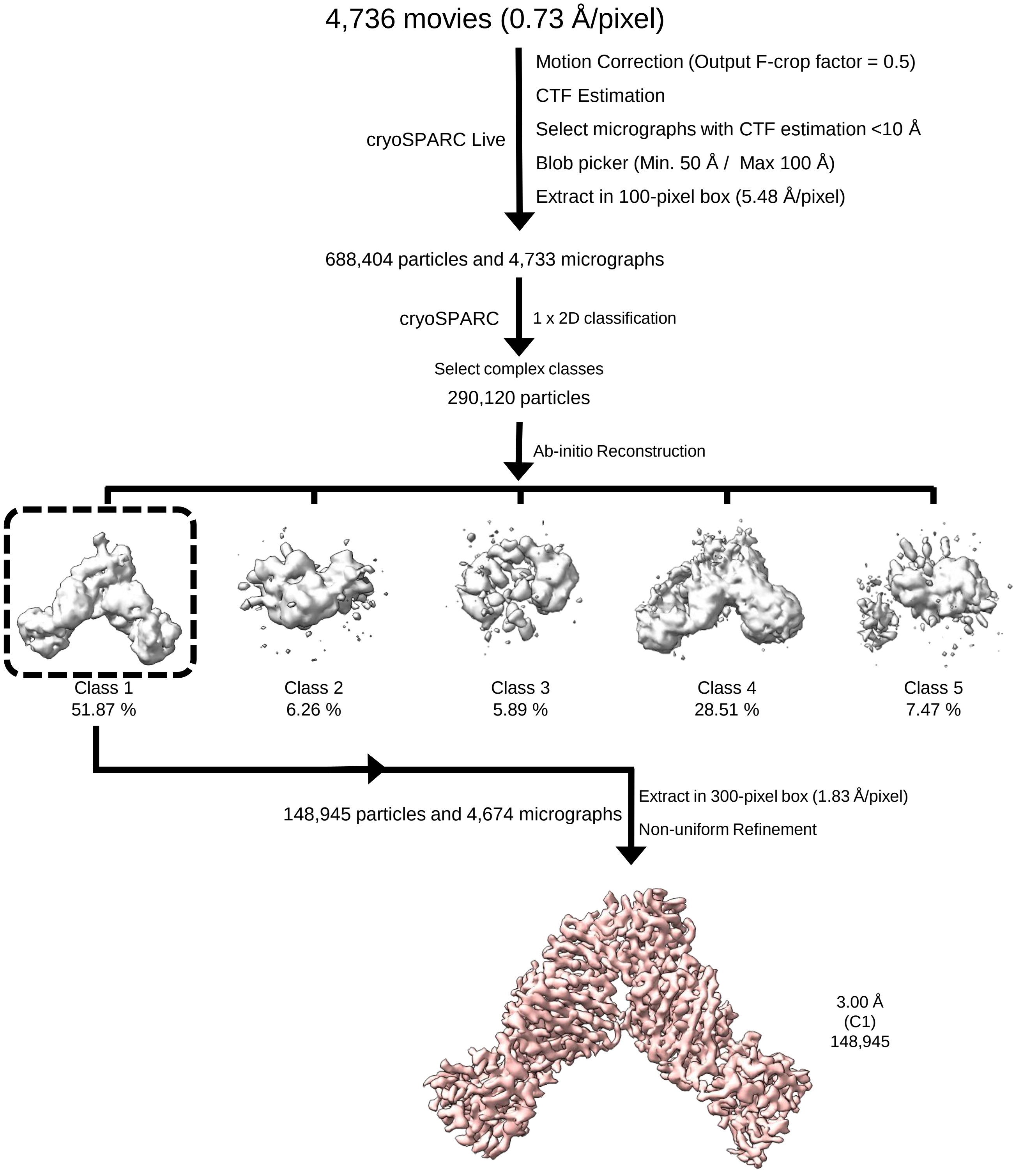

Fig.S9 Single-particle cryo-EM data processing pipeline for the PDCoV 67B12/42H3 Fab complex.

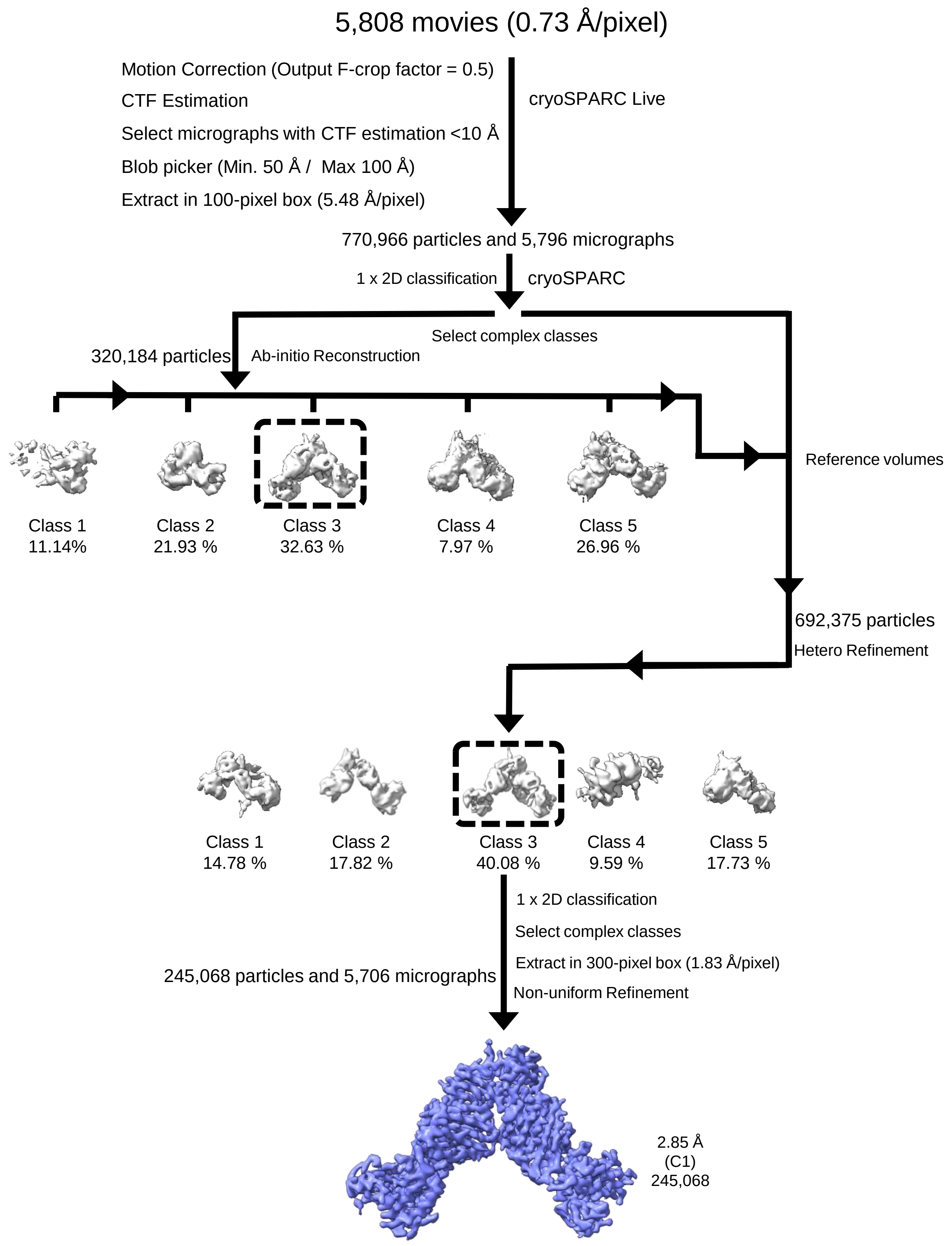

Fig.S10 Single-particle cryo-EM data processing pipeline for the PDCoV S1B-67B12/46E6 Fab complex.

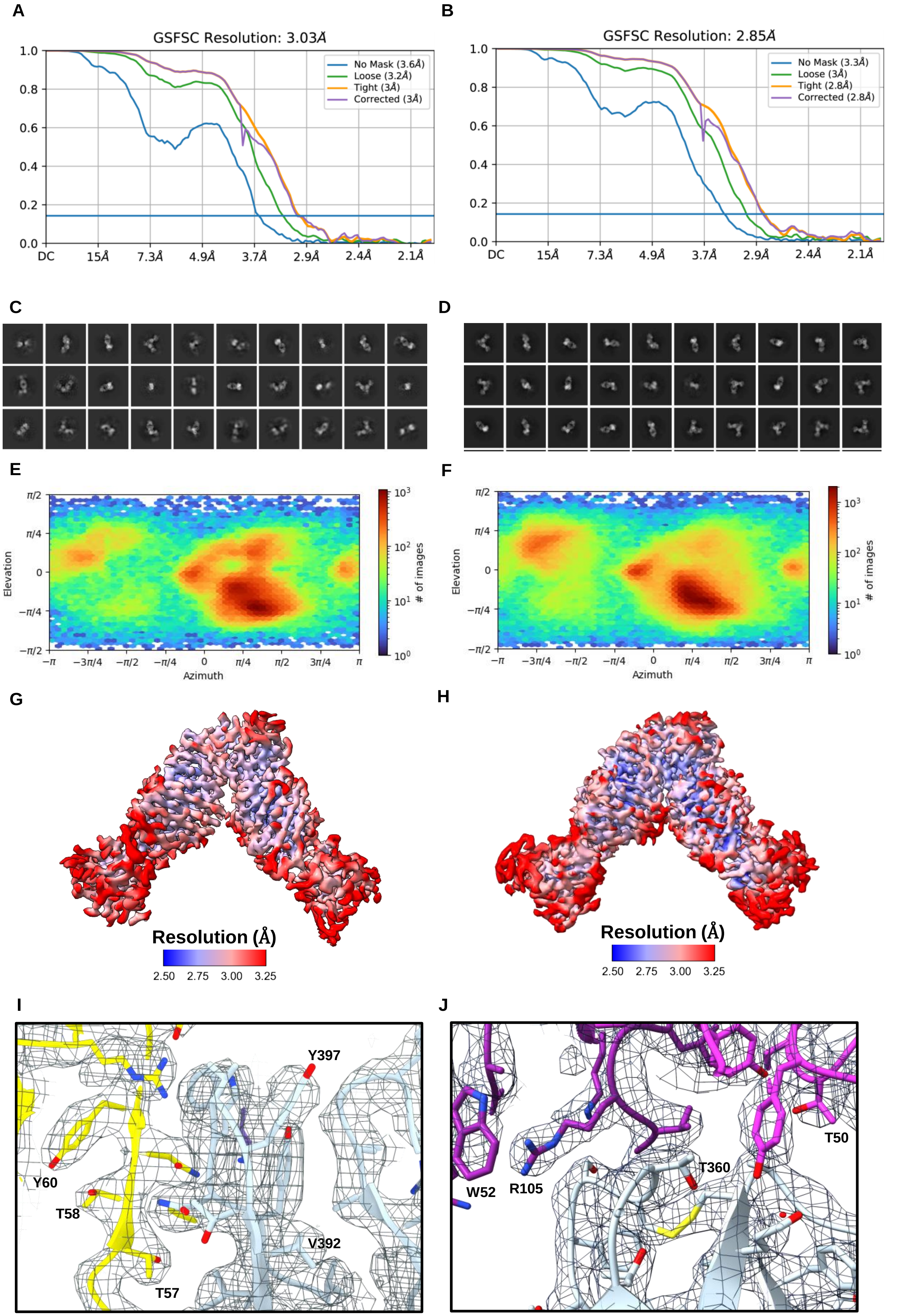
A
GSFSC Resolution: 3.03Å
No Mask (3.6Å)
Loose (3.2Å)
Tight (3Å)
Corrected (3Å)
DC 15Å 7.3Å 4.9Å 3.7Å 2.9Å 2.4Å 2.1Å
B
GSFSC Resolution: 2.85Å
No Mask (3.3Å)
Loose (3Å)
Tight (2.8Å)
Corrected (2.8Å)
C
D
E
F
Elevation
Azimuth
# of images
G
H
Resolution (Å)
2.50 2.75 3.00 3.25
I
Y397
Y60
T58
V392
T57
J
T50
W52
R105
T360

Fig.S11 Single-particle cryo-EM data processing for the PDCoV S S1B-67B12/42H3 & S1B-67B12/46E6 Fab complexes. (**A**) Gold-standard Fourier shell correlation (FSC) curve generated from the independent half maps contributing to the 3.0 Å global resolution density map of the PDCoV S1B in complex with 67B12 and 42H3 Fab fragments. (**B**) As shown in A for the 2.8 Å global resolution density map of the PDCoV S1B in complex with 67B12 and 46E6 Fab fragments. (**C**) Representative 2D classes for PDCoV S S1B-67B12/42H3. (**D**) Representative 2D classes for PDCoV S S1B-67B12/46E6. (**E**) Angular distribution plot of the final for PDCoV S S1B-67B12/42H3 C1 refined EM density maps. (**F**) Angular distribution plot of the final for PDCoV S S1B-67B12/46E6 C1 refined EM density maps. (**G**) Local resolution filtered EM density map for the refined PDCoV S1B in complex with 67B12 and 42H3 Fab fragments, colored according to local resolution which was calculated in CryoSPARC. (**H**) As shown in C for the PDCoV S1B in complex with 67B12 and 46E6 Fab fragments. **(I)** Zoomed-in view of the interacting region of 67B12 and PDCoV S1B with the EM density of complex 1 shown as a black mesh. **(J)** Zoomed-in view of the interacting region of 46E6 and PDCoV S1B with the EM density of complex 2 shown as a black mesh.

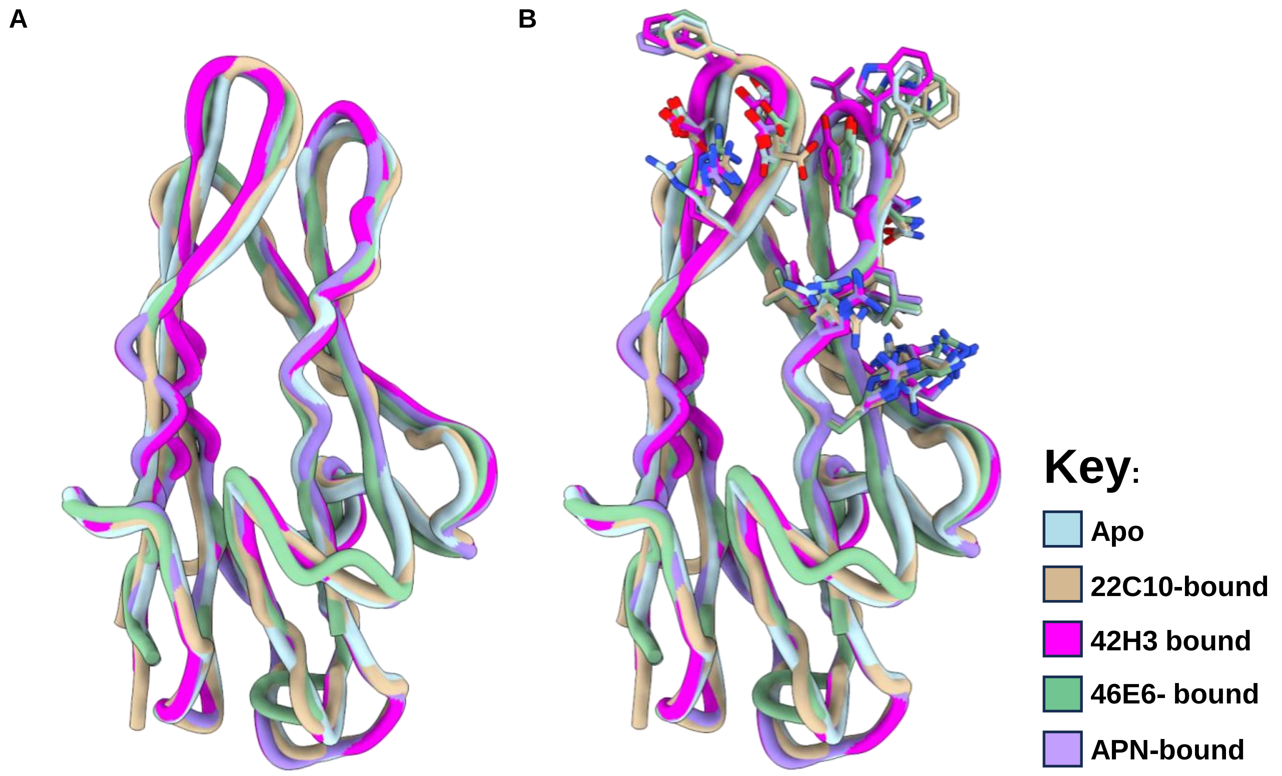

Fig.S12 Structural investigation of 42H3/46E6 binding on PDCoV S1B. (**A**) Comparison of the PDCoV S1B monomer during different interactions. (**B**) Comparison of the PDCoV S1B monomer during different interactions, showing all atoms that interact with human APN. Apo structure, 22C10-bound structure, 42H3-bound structure, 46E6-bound structure and APN-bound structure are depicted in blue, tan, magenta, green and purple, respectively.

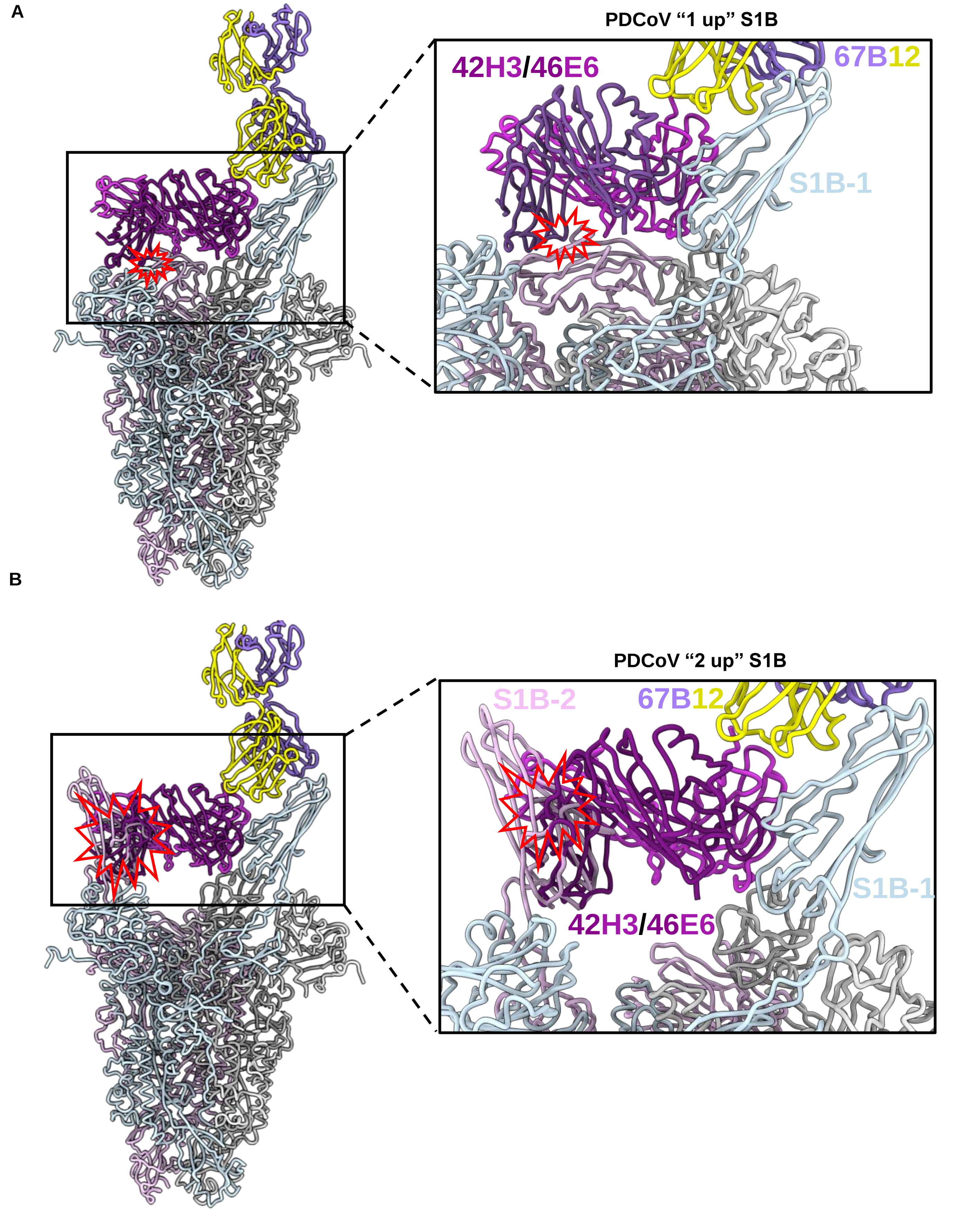

Fig.S13 Structural representation of potential S1 shedding mechanism induced by 42H3/46E6 binding. (**A**) Hypothetical PDCoV spike with “1 up” S1B monomer, made using the PEDV CTD open conformation structure (PDB: 7Y6V), superimposed onto the trimeric 22C10-bound PDCoV structure, along with complex 1 (67B12 and 42H3-bound S1B). (**B**) Hypothetical PDCoV spike with “2 up” S1B monomers, made using 2 copies of the PEDV CTD open conformation structure (PDB: 7Y6V), superimposed onto the trimeric 22C10-bound PDCoV structure, along with complex 1 (67B12 and 42H3-bound S1B). Clashes which may induce steric hinderance are indicated with a red clash sign.

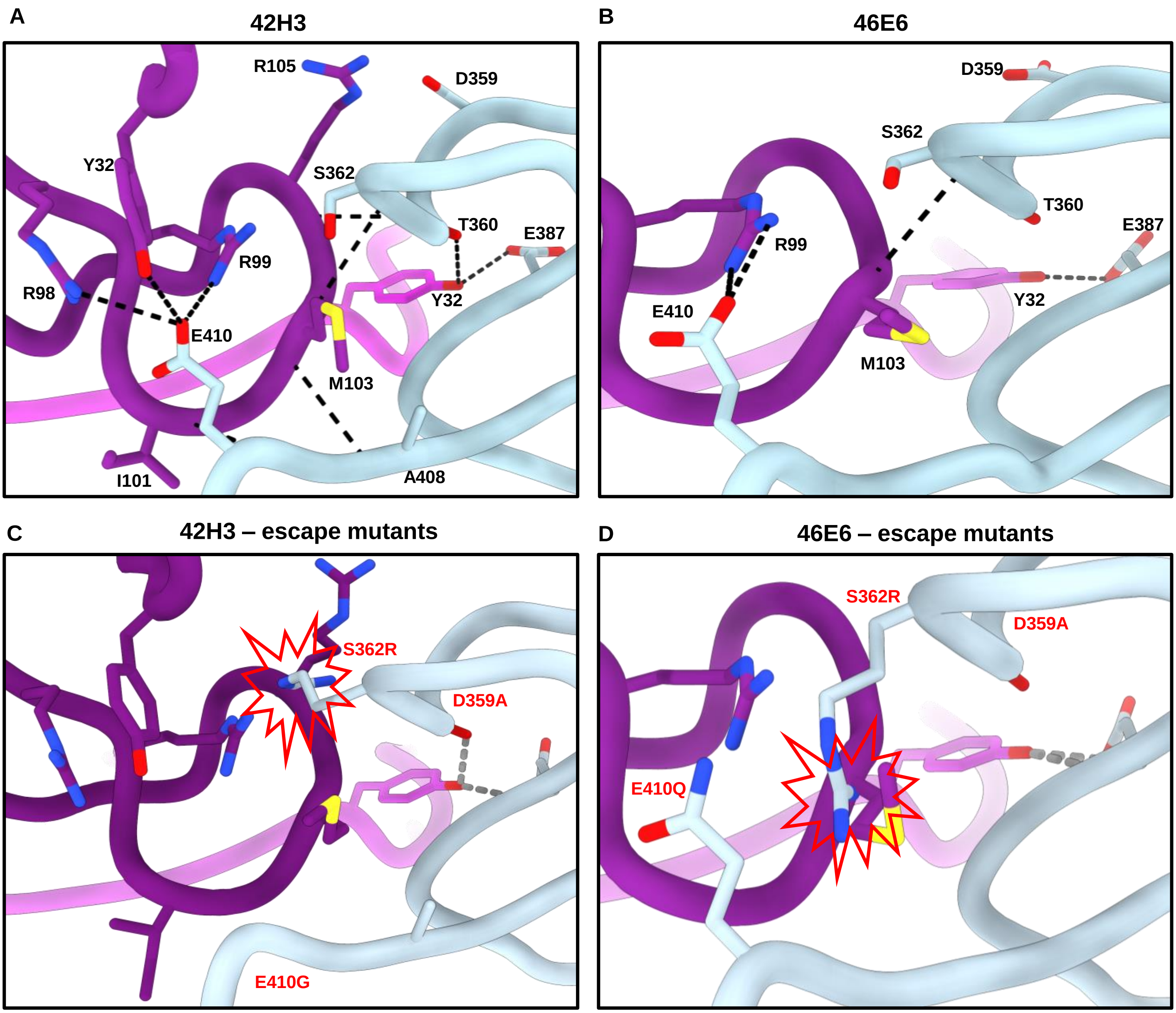

Fig.S14 Structural representation of escape mutants against 42H3 and 46E6. (**A**) Observed interactions between PDCoV S1B and 42H3. (**B**) Observed interactions between PDCoV S1B and 46E6. (**C**) Structural representation of PDCoV S1B escape mutants isolated in serial passage experiments in the context of the 42H3 interaction. (**D**) Structural representation of PDCoV S1B escape mutants isolated in serial passage experiments in the context of the 46E6 interaction. Mutated amino acids are shown, with mutation labelled in red. Clashes which may induce steric hinderance are indicated with a red clash sign.

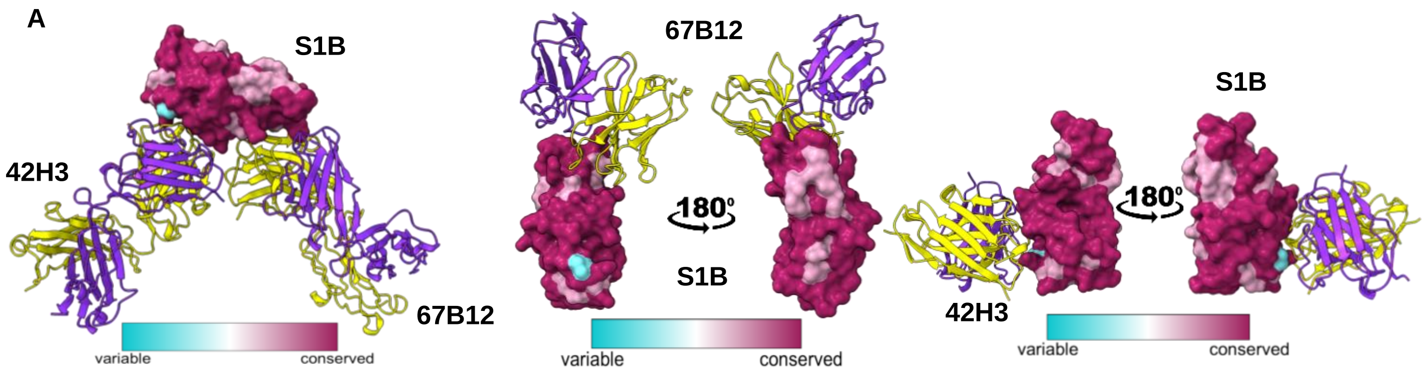

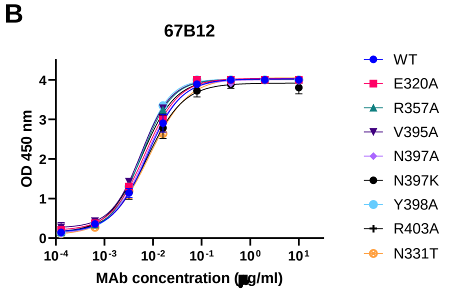

Fig.S15 (**A**) Mapping of PDCoV amino acid conservation onto the surface representation of PDCoV S1B domain in complex with the 42H3 and 67B12 Fab fragments. (**B**) ELISA binding reactivity of 67B12 to PDCoV S1 variants carrying the indicated mutations. Results represent the mean (±SD) from two independent experiments.

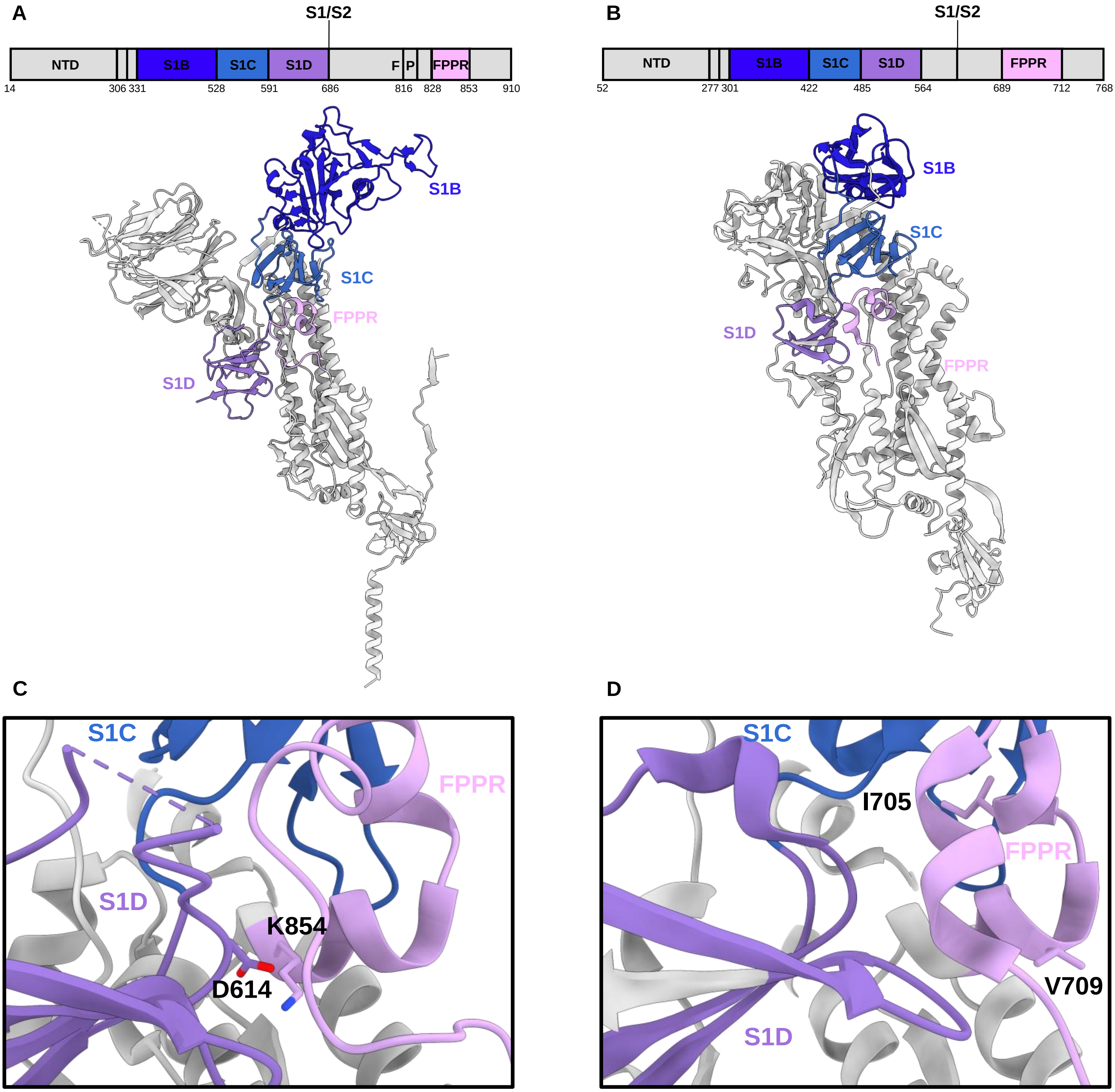

Fig.S16 Cryo-EM structures of the SARS-CoV-2 and PDCoV S protein. (**A**) (Top) The structure of the closed, three RBD–down conformation of the D614 SARS-CoV-2 S is shown in ribbon diagram with one protomer colored as, S1B in purple, S1C in blue, S1D in violet, and the FPPR in pink. (Bottom) A close-up view of the region near the residue 614 with colored as, S1B in purple, S1C in blue, S1D in violet, and the FPPR in pink. Residues D614 and K854 are shown in stick model. (**B**) (Top) The structure of the closed, three RBD–down conformation of the PDCoV S is shown in ribbon diagram with one protomer colored as, S1B in purple, S1C in blue, S1D in violet, and the FPPR in pink. (Bottom) A close-up view of the region near the residue 614 with colored as, S1B in purple, S1C in blue, S1D in violet, and the FPPR in pink. Escape mutant residues I705 and V709 are shown in stick model.

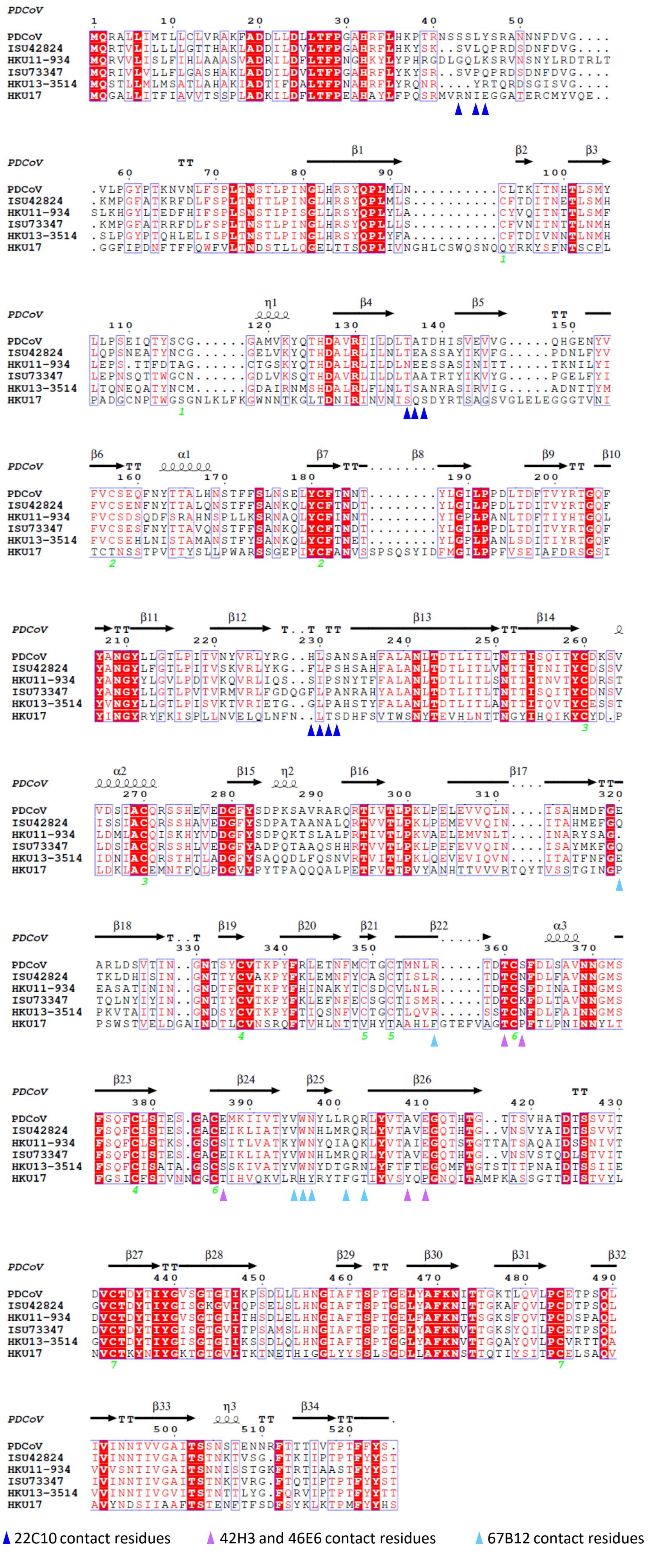
22C10 contact residues
42H3 and 46E6 contact residues
67B12 contact residues

Fig.S17 Sequence alignment of the S protein S1 region from various deltacoronaviruses. The S1 protein sequence of PDCoV was aligned with those of sparrow coronaviruses (HKU17, ISU42824 and ISU73347), munia coronavirus (HKU13-3514), and bulbul coronavirus (HKU11-934). The sequence alignment including secondary structure elements based on the PDCoV S cryoEM structure (PDB: 6BFU) was generated with ESPript 3.0. Antibody contact residues - determined for PDCoV S based on PDBePISA and Ligplot predictions - are indicated with arrowheads under the alignment.

Table.S1

Cryo-EM data collection, refinement and validation statistics

	PDCoV-22C10 (Global) (EMDB-19014) (PDB 8R9W)	PDCoV-22C10 (Local) (EMDB- 19015) (PDB 8R9X)	PDCoV-Complex1 (EMDB-19016) (PDB 8R9Y)	PDCoV-Complex2 (EMDB-19017) (PDB 8R9Z)
**Data collection and processing**				
Magnification	165,000	165,000	165,000	165,000
Voltage (kV)	300	300	300	300
Electron exposure (e–/Å^2)	50	50	50	50
Defocus range (μm)	-0.75 to -1.5	-0.75 to -1.5	-0.75 to -1.5	-0.75 to -1.5
Pixel size (Å)	0.73	0.73	0.73	0.73
Symmetry imposed	C3	C1	C1	C1
Initial particle images (no.)	533,609	533,609	688,404	770,966
Final particle images (no.)	168,789	112,743	148,945	245,068
Map resolution (Å)	3.0	3.1	3.0	2.9
FSC threshold	0.143	0.143	0.143	0.143
Map resolution range (Å)				
**Refinement**				
Initial model used (PDB code)	6BFU	6BFU	6BFU	6BFU
Model resolution (Å)	3.1	3.1	3.0	3.1
FSC threshold	0.5	0.5	0.5	0.5
Map sharpening *B* factor (Å^2)	85.6	94.0	75.2	80.6
Model composition				
Non-hydrogen atoms	26331	3782	7448	7443
Protein residues	3540	461	972	972
Ligands	BMA: 6<br>NAG: 63<br>MAN: 6	BMA:1<br>NAG: 11<br>FUC: 1<br>MAN: 2	NAG: 3	NAG: 3
B factors (Å^2)				
Protein	140.7	54.6	104.9	74.3
Ligand	141.6	69.6	129.3	91.9
R.m.s. deviations				
Bond lengths (Å)	0.003 (3)	0.004 (0)	0.003 (0)	0.003 (0)
Bond angles (°)	0.580 (3)	0.650 (0)	0.578 (1)	0.561 (0)
Validation				
MolProbity score	1.61	1.58	1.68	1.66
Clashscore	5.04	5.12	5.97	5.23
Poor rotamers (%)	0.72	0.00	0.00	0.00
Ramachandran plot				
Favored (%)	95.03	95.60	94.89	94.36
Allowed (%)	4.97	4.40	5.11	5.64
Disallowed (%)	0.00	0.00	0.00	0.00
