# Supplementary material for: Neutralizing antibodies reveal cryptic vulnerabilities and interdomain crosstalk in the porcine deltacoronavirus spike protein
Source: Nat Commun. 2024 Jun 22;15:5330. doi: 10.1038/s41467-024-49693-0 (PMC11193727; doi:10.1038/s41467-024-49693-0)
Supplement: Supplementary file 3 — Reporting Summary [file 41467_2024_49693_MOESM3_ESM.pdf]

nature portfolio

Corresponding author(s): Berend-Jan Bosch

Last updated by author(s): 22-12-2023

# Reporting Summary

Nature Portfolio wishes to improve the reproducibility of the work that we publish. This form provides structure for consistency and transparency in reporting. For further information on Nature Portfolio policies, see our Editorial Policies and the Editorial Policy Checklist.

## Statistics

For all statistical analyses, confirm that the following items are present in the figure legend, table legend, main text, or Methods section.

| n/a | Confirmed | |
|---|---|---|
| ☐ | ☒ | The exact sample size (*n*) for each experimental group/condition, given as a discrete number and unit of measurement |
| ☐ | ☒ | A statement on whether measurements were taken from distinct samples or whether the same sample was measured repeatedly |
| ☒ | ☐ | The statistical test(s) used AND whether they are one- or two-sided<br>*Only common tests should be described solely by name; describe more complex techniques in the Methods section.* |
| ☒ | ☐ | A description of all covariates tested |
| ☒ | ☐ | A description of any assumptions or corrections, such as tests of normality and adjustment for multiple comparisons |
| ☐ | ☒ | A full description of the statistical parameters including central tendency (e.g. means) or other basic estimates (e.g. regression coefficient) AND variation (e.g. standard deviation) or associated estimates of uncertainty (e.g. confidence intervals) |
| ☒ | ☐ | For null hypothesis testing, the test statistic (e.g. *F*, *t*, *r*) with confidence intervals, effect sizes, degrees of freedom and *P* value noted<br>*Give P values as exact values whenever suitable.* |
| ☒ | ☐ | For Bayesian analysis, information on the choice of priors and Markov chain Monte Carlo settings |
| ☒ | ☐ | For hierarchical and complex designs, identification of the appropriate level for tests and full reporting of outcomes |
| ☒ | ☐ | Estimates of effect sizes (e.g. Cohen's *d*, Pearson's *r*), indicating how they were calculated |

*Our web collection on statistics for biologists contains articles on many of the points above.*

## Software and code

Policy information about availability of computer code

| | |
|---|---|
| Data collection | ELISA plate reader (EL-808, Biotek) was used to collect ELISA data. EVOS FL fluorescence microscope (Thermo Fisher Scientific, the Netherlands) was used to collect fluorescence images. The Octet (Octet RED384, Fortebio) was used to collect affinity data and epitope binning data. Titan Krios (Thermo Fisher Scientific) was used to collect cryo-EM data. Online protein sequence alignment tool: http://www/ebi/ac/uk/Tooll/msa/clustalo/. |
| Data analysis | GraphPad Prism (version 8), Fortebio Data Analysis 7.0, ImageJ,CryoSPARC v4, UCSF Chimera 1.15.0, SBGrid, PDBePISA 1.52, LigPlot+ 2.2, PLGS 3.0.1, Phenix 1.19.2_4158, UCSF ChimeraX 1.2.5. |

For manuscripts utilizing custom algorithms or software that are central to the research but not yet described in published literature, software must be made available to editors and reviewers. We strongly encourage code deposition in a community repository (e.g. GitHub). See the Nature Portfolio guidelines for submitting code & software for further information.

## Data

Policy information about availability of data

All manuscripts must include a data availability statement. This statement should provide the following information, where applicable:

- Accession codes, unique identifiers, or web links for publicly available datasets
- A description of any restrictions on data availability
- For clinical datasets or third party data, please ensure that the statement adheres to our policy

The PDB file of PDCoV spike protein (PDB ID: 6BFU) and SARS-CoV-2 spike protein (PDB ID: 6XR8) were downloaded from NCBI database (https://www.ncbi.nlm.nih.gov/). Spike protein sequences used in this study were downloaded from NCBI database (https://www.ncbi.nlm.nih.gov/) (See Supplementary Figure 6 for the accession numbers). The cryo-EM maps and atomic structures have been deposited in the Protein Data Bank (PDB) Electron Microscopy Data Bank (EMDB) under accession codes: 8R9W and EMD-19014 for PDCoV S/22C10 Fab complex (global), 8R9X and EMD-19015 for PDCoV S/22C10 Fab complex (local refinement), 8R9Y and EMD-19016 for PDCoV S1B-67B12/42H3 Fab complex, and 8R9Z and EMD-19017 for PDCoV S1B-67B12/46E6 Fab complex. Source data will be provided with this paper.

## Research involving human participants, their data, or biological material

Policy information about studies with human participants or human data. See also policy information about sex, gender (identity/presentation), and sexual orientation and race, ethnicity and racism.

| | |
|---|---|
| Reporting on sex and gender | n.a. |
| Reporting on race, ethnicity, or other socially relevant groupings | n.a. |
| Population characteristics | n.a. |
| Recruitment | n.a. |
| Ethics oversight | n.a. |

Note that full information on the approval of the study protocol must also be provided in the manuscript.

# Field-specific reporting

Please select the one below that is the best fit for your research. If you are not sure, read the appropriate sections before making your selection.

☒ Life sciences ☐ Behavioural & social sciences ☐ Ecological, evolutionary & environmental sciences

For a reference copy of the document with all sections, see nature.com/documents/nr-reporting-summary-flat.pdf

# Life sciences study design

All studies must disclose on these points even when the disclosure is negative.

| | |
|---|---|
| Sample size | For infection experiments, 50,000 cells were taken per sample. Infection dose of authentic virus infection experiments was based on MOI to prevent double infections of cells. The sample sizes were sufficient for a good statistical analysis based on previous experience (PMID: 32366817). |
| Data exclusions | No data were excluded from the analyses. |
| Replication | Experimental findings were reliably reproduced. Most of the experiments were replicated two or three times, as noted in the figure legends and text. |
| Randomization | All samples were allocated in random order, no particular bias is envisaged. |
| Blinding | Blinding was not relevant as the reported data was not based on subjective observations, but quantitative measurements, including ELISA etc. |

# Reporting for specific materials, systems and methods

We require information from authors about some types of materials, experimental systems and methods used in many studies. Here, indicate whether each material, system or method listed is relevant to your study. If you are not sure if a list item applies to your research, read the appropriate section before selecting a response.

## Materials & experimental systems

| n/a | Involved in the study | |
|---|---|---|
| ☐ | ☒ | Antibodies |
| ☐ | ☒ | Eukaryotic cell lines |
| ☒ | ☐ | Palaeontology and archaeology |
| ☒ | ☐ | Animals and other organisms |
| ☒ | ☐ | Clinical data |
| ☒ | ☐ | Dual use research of concern |
| ☒ | ☐ | Plants |

## Methods

| n/a | Involved in the study | |
|---|---|---|
| ☒ | ☐ | ChIP-seq |
| ☒ | ☐ | Flow cytometry |
| ☒ | ☐ | MRI-based neuroimaging |

# Antibodies

Antibodies used

1. StrepMAB-Classic, HRP conjugate, Cat.no: 2-1509-001, IBA, 1:2,000
2. Donkey anti-Mouse IgG (H+L) Cross-Adsorbed Secondary Antibody Alexa Fluor 488, Catalog # A-21202, Invitrogen, 1:400
3. Goat anti-human IgG (HRP), Catalog #2040-05, ITK Southern Biotech, 1:2000
4. Mouse anti-rat IgG1/2b/2c, Catalog # KT96/KT98/KT99, Absea Biotechnology Ltd., 1:2000
5. Goat anti-human Alexa Fluor 488 antibody, Catalog# A-11013, Invitrogen,1:400
6. PDCoV nucleocapsid antibodies,Catalog # PDCOV11-M, Clinisciences,1:2000
7. Anti-streptag monoclonal antibody (PMID: 30938227)
8. PDCoV S specific antibodies isolated from humanized H2L2 mice (PMID: 33731724)
9.HRP-conjugated goat anti-mouse IgG, Catalog# 31432, Invitrogen

Validation

Validation of the PDCoV S specific antibodies was done by ELISA binding assays, antibody binding competition assays and virus neutralization assays, as detailed in this study. Validation of anti-streptag isotype control antibody was described earlier (PMID: 30938227). All the secondary antibodies were bought from commercial vendors and were validated by the manufacturers.

# Eukaryotic cell lines

Policy information about cell lines and Sex and Gender in Research

Cell line source(s)

Human embryonic kidney cell, HEK293T, from ATCC® CRL-11268
Huh7 cell, from Japanese Collection of Research Bioresources (JCRB0403)

Authentication

The cell lines were not authenticated further after purchase.

Mycoplasma contamination

We confirm that all cells were tested as mycoplasma negative.

Commonly misidentified lines
(See ICLAC register)

No commonly misidentified cell lines were used.

# Plants

Seed stocks

n.a.

Novel plant genotypes

n.a.

Authentication

n.a.
